# Correction for Hiramatsu et al., “The Mechanism of Pertussis Cough Revealed by the Mouse-Coughing Model”

**DOI:** 10.1128/mbio.01446-22

**Published:** 2022-05-31

**Authors:** Yukihiro Hiramatsu, Koichiro Suzuki, Takashi Nishida, Naoki Onoda, Takashi Satoh, Shizuo Akira, Masahito Ikawa, Hiroko Ikeda, Junzo Kamei, Sandra Derouiche, Makoto Tominaga, Yasuhiko Horiguchi

**Affiliations:** a Department of Molecular Bacteriology, Research Institute for Microbial Diseases, Osaka University, Suita, Osaka, Japan; b The Research Foundation for Microbial Diseases of Osaka University (BIKEN), Suita, Osaka, Japan; c Laboratory of Host Defense, World Premier Institute Immunology Frontier Research Center, Osaka University, Suita, Osaka, Japan; d Department of Experimental Genome Research, Research Institute for Microbial Diseases, Osaka University, Suita, Osaka, Japan; e Department of Pathophysiology and Therapeutics, Hoshi University School of Pharmacy and Pharmaceutical Sciences, Tokyo, Japan; f Juntendo Advanced Research Institute for Health Science, Juntendo University, Tokyo, Japan; g Division of Cell Signaling, National Institute for Physiological Sciences, Okazaki, Aichi, Japan; h Thermal Biology Group, Exploratory Research Center on Life and Living Systems (ExCELLS), Okazaki, Aichi, Japan; i Center for Infectious Disease Education and Research, Osaka University, Suita, Osaka, Japan

## AUTHOR CORRECTION

Volume 13, no. 2, e03197-21, 2022, https://doi.org/10.1128/mbio.03197-21. In the Discussion section, we additionally cite the following article that previously discussed the possibility that Vag8 and Bdk are involved in pertussis cough: E. S. Hovingh, S. de Maat, A. P. M. Cloherty, S. Johnson, et al., Front Immunol 9:1172, 2018, https://doi.org/10.3389/fimmu.2018.01172.

In the legend to Fig. S4, the correct reference for BP141 is as follows, which can be found in the reference list of Table S2: N. Otsuka, H. J. Han, H. Toyoizumi-Ajisaka, Y. Nakamura, et al., PLoS ONE 7:e31985, 2012, https://doi.org/10.1371/journal.pone.0031985.

